# Diagnosis, clinical assessment, and staging of hepatocellular carcinoma: a Brazilian multidisciplinary consensus

**DOI:** 10.1590/0102-672020260000035e1964

**Published:** 2026-08-31

**Authors:** Felipe José Fernández COIMBRA, André Luís de GODOY, Paulo Henrique de Sousa FERNANDES, Heládio FEITOSA, Rodrigo Nascimento PINHEIRO, Ilka de Fátima Santana Ferreira BOIN, Wellington ANDRAUS, Francisco TUSTUMI, Paulo Cezar Galvão do AMARAL, Carlos Eduardo BRANDÃO MELLO, Alexcia Camila BRAUN, Maria Ignez BRAGHIROLI, Luis César BREDT, Carla CAPARROZ, Flair José CARRILHO, Guilherme Moura CUNHA, Alessandro Landskron DINIZ, Daniel Neves FORTE, José Huygens Parente GARCIA, Tércio GENZINI, Mauro Monteiro CORREIA, Cássio Virgílio Cavalcante de OLIVEIRA, Antonio Nocchi KALIL, Agnaldo Soares LIMA, Guilherme Lopes Pinheiro MARTINS, Cristina Melo ROCHA, Marcos Roberto de MENEZES, Joaquim Maurício da MOTTA-LEAL-FILHO, Munir MURAD, Renata D’Alpino PEIXOTO, Rafael Soares PINHEIRO, Marcelo Bruno REZENDE, Héber Salvador de Castro RIBEIRO, Mario Rino MARTINS, Duilio Reis da ROCHA, Arthur Accioly ROSA, Luiz Arnaldo SZUTAN, Orlando Jorge Martins TORRES, Luiz Augusto CARNEIRO D’ALBUQUERQUE, Ângelo Alves de MATTOS, Anelisa Kruschewsky COUTINHO, Paulo HERMAN, Alexandre Ferreira OLIVEIRA

**Affiliations:** 1 A.C. Camargo Cancer Center, HPB & Upper GI Oncology Reference Center, Abdominal Surgery, Department of Surgical Oncology - São Paulo (SP), Brazil.; 2 Universidade Federal de Uberlândia, Department of Surgical Oncology - Uberlândia (MG), Brazil.; 3 Instituto do Câncer do Ceará, Department of Surgical Oncology - Fortaleza (CE), Brazil.; 4 Hospital de Base do Distrito Federal, Department of Surgical Oncology - Brasília (DF), Brazil.; 5 Universidade Estadual de Campinas, Department of Surgery - Campinas (SP), Brazil.; 6 Universidade de São Paulo, Department of Gastroenterology - São Paulo (SP), Brazil.; 7 São Rafael Hospital/Rede D’Or Hospital Group, Department of Surgery of the Upper Digestive System - Salvador (BA), Brazil.; 8 Universidade Federal do Estado do Rio de Janeiro, Department of Internal Medicine - Rio de Janeiro (RJ), Brazil.; 9 Instituto do Câncer do Estado de São Paulo, Department of Oncology - São Paulo (SP), Brazil.; 10 Universidade Estadual do Oeste do Paraná, Department of Surgical Oncology and Hepatobiliary Surgery - Cascavel (PR), Brazil.; 11 Hospital Beneficência Portuguesa de São Paulo, Department of Radiology - São Paulo (SP), Brazil.; 12 University of Washington, Department of Radiology - Seattle, United States of America.; 13 Universidade de São Paulo, Emergency Department - São Paulo (SP), Brazil.; 14 Universidade Federal do Ceará, Walter Cantídio University Hospital, Liver Transplant Unit, Department of Surgery - Fortaleza (CE), Brazil.; 15 Leforte Hospital, Pancreas and Kidney Transplant Program - São Paulo (SP), Brazil.; 16 Instituto Nacional de Câncer, Department of Abdominopelvic Surgery - Rio de Janeiro (RJ), Brazil.; 17 Universidade Federal da Paraíba, Department of Surgery - João Pessoa (PB), Brazil.; 18 Irmandade Santa Casa de Misericórdia de Porto Alegre, Department of Hepato-Biliary-Pancreatic Surgery and Liver Transplantation - Porto Alegre (RS), Brazil.; 19 Universidade Federal de Minas Gerais, Department of Surgery - Belo Horizonte (MG), Brazil.; 20 Instituto do Câncer do Estado de São Paulo, Department of Radiology - São Paulo (SP), Brazil.; 21 Universidade Federal do Amazonas, Department of Gastroenterology - Manaus (AM), Brazil.; 22 Universidade de São Paulo, Department of Radiology - São Paulo (SP), Brazil.; 23 Universidade Federal de Minas Gerais, Department of Oncology - Belo Horizonte (MG), Brazil.; 24 University of British Columbia, Department of Medical Oncology - Vancouver, Canada.; 25 Hospital Israelita Albert Einstein, Department of Health Sciences - São Paulo (SP), Brazil.; 26 Hospital de Câncer de Pernambuco, Department of Oncological Surgery - Recife (PE), Brazil.; 27 Universidade Federal do Ceará, Department of Oncology - Fortaleza (CE), Brazil.; 28 Oncoclínicas Salvador and Hospital Santa Izabel, Department of Radiation Oncology - Salvador (BA), Brazil.; 29 Faculdade de Ciências Médicas da Santa Casa de São Paulo, Department of Surgery - São Paulo (SP), Brazil.; 30 Universidade Federal do Maranhão, Department of Gastrointestinal Surgery - São Luís (MA), Brazil.; 31 Universidade Federal de Ciências da Saúde de Porto Alegre, Department of Gastroenterology and Hepatology - Porto Alegre (RS), Brazil.; 32 Rede Mater Dei, Department of Oncology - Salvador (BA), Brazil.; 33 Universidade Federal de Juiz de Fora, Department of Surgery - Juiz de Fora (MG), Brazil.

**Keywords:** Carcinoma, Hepatocellular. Consensus. Guidelines as Topic. Diagnosis. Magnetic Resonance Imaging. Biopsy, Carcinoma Hepatocelular, Consenso, Guias como Assunto, Diagnóstico, Imageamento por Ressonância Magnética, Biópsia

## Abstract

**Background:**

Hepatocellular carcinoma (HCC) is a leading cause of cancer-related mortality worldwide, with a rising incidence largely driven by chronic liver disease. Accurate diagnosis and appropriate clinical assessment at the time of presentation are essential, as therapeutic strategies and prognosis depend on tumor burden, liver function, portal hypertension, and patient performance status.

**Aim:**

To develop evidence-based, multidisciplinary recommendations to guide the diagnosis, clinical assessment, and staging of patients with HCC.

**Methods:**

This consensus was developed by 43 experts from surgical oncology, hepatology, clinical oncology, radiology, interventional radiology, pathology, liver transplantation, gastroenterology, radiation oncology, and palliative care, under the coordination of the Brazilian Society of Surgical Oncology and 13 collaborating national medical societies. A scientific steering committee predefined clinically relevant questions addressing radiological and histopathological diagnosis, clinical assessment, diagnostic work-up, management of patients at the time of HCC diagnosis, and staging. These questions were discussed and refined in multidisciplinary meetings and submitted to structured voting rounds.

**Results:**

The panel formulated 18 recommendations covering key aspects of HCC evaluation, including standardized application of Liver Imaging Reporting and Data System (LI-RADS^®^) for imaging-based diagnosis, management of indeterminate lesions, indications for biopsy, histopathological classification and reporting, immunohistochemical markers, assessment of hepatic function and portal hypertension, staging systems, diagnostic work-up, and the role of multidisciplinary care. The recommendations emphasize integration of imaging findings with liver-related factors and clinical context to support individualized decision-making.

**Conclusions:**

This multidisciplinary consensus provides practical, evidence-based recommendations for the diagnosis, clinical assessment, and staging of HCC. By promoting standardized diagnostic practices while reinforcing comprehensive patient evaluation and multidisciplinary management, this document aims to improve diagnostic accuracy, optimize treatment selection, and support safe care.

## ARTICLE HIGHLIGHTS

• This work is part of a national, multidisciplinary series of Brazilian consensus documents on hepatocellular carcinoma.

• A total of 43 experts from multiple Brazilian medical societies and specialties participated in the consensus process.

• The document provides evidence-based recommendations focused on diagnosis, clinical assessment, and staging of hepatocellular carcinoma.

## Summary

**CENTRAL MESSAGE:**

This document is part of a national, multidisciplinary consensus series on hepatocellular carcinoma in Brazil. Developed by 43 experts from 14 medical societies, it focuses on the diagnosis, clinical assessment, and staging of hepatocellular carcinoma. The goal is to provide practical evidence-based recommendations.

**PERSPECTIVES:**

This consensus aims to improve and standardize the strategies for the diagnosis, clinical assessment, and staging of hepatocellular carcinoma. By providing clear recommendations, the document seeks to reduce unnecessary tests and biopsies, enhance diagnostic accuracy, improve initial patient care after diagnosis of hepatocellular carcinoma, and support more efficient clinical decision-making.

## VISUAL ABSTRACT

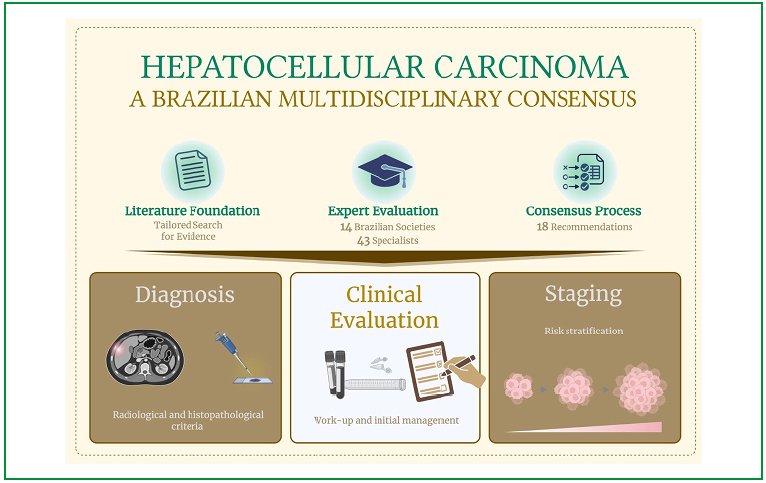

## RESUMO

**Racional:**

O carcinoma hepatocelular (CHC) é uma das principais causas de mortalidade por câncer em todo o mundo, com incidência crescente, impulsionada principalmente por doenças hepáticas crônicas. O diagnóstico preciso e a avaliação clínica adequada no momento da apresentação são essenciais, uma vez que as estratégias terapêuticas e o prognóstico dependem da carga tumoral, da função hepática, da hipertensão portal e do estado geral do paciente.

**Objetivo:**

Desenvolver recomendações multidisciplinares baseadas em evidências para orientar o diagnóstico, a avaliação clínica e o estadiamento de pacientes com CHC.

**Métodos:**

Este consenso foi desenvolvido por 43 especialistas em oncologia cirúrgica, hepatologia, oncologia clínica, radiologia, radiologia intervencionista, patologia, transplante hepático, gastroenterologia, radiooncologia e cuidados paliativos, sob a coordenação da Sociedade Brasileira de Oncologia Cirúrgica e 13 sociedades médicas nacionais colaboradoras. Um comitê científico-diretor predefiniu questões clinicamente relevantes, abrangendo o diagnóstico radiológico e histopatológico, a avaliação clínica, a investigação diagnóstica, o manejo dos pacientes no momento do diagnóstico de CHC e o estadiamento. Essas questões foram discutidas e refinadas em reuniões multidisciplinares e submetidas a rodadas de votação estruturadas.

**Resultados:**

O painel formulou 18 recomendações que abrangem aspectos-chave da avaliação do CHC, incluindo a aplicação do Liver Imaging Reporting and Data System (LI-RADS^®^; sistema padronizado para interpretação e relato de exames de imagem hepática) para diagnóstico, manejo de lesões indeterminadas, indicações para biópsia, classificação e relato histopatológico, marcadores imuno-histoquímicos, avaliação da função hepática e hipertensão portal, sistemas de estadiamento, investigação diagnóstica e o papel do atendimento multidisciplinar. As recomendações enfatizam a integração dos achados de imagem com fatores relacionados ao fígado e o contexto clínico para apoiar a tomada de decisão individualizada.

**Conclusões:**

Este consenso multidisciplinar fornece recomendações práticas e baseadas em evidências para o diagnóstico, a avaliação clínica e o estadiamento do CHC. Ao promover práticas diagnósticas padronizadas, reforçar a avaliação abrangente do paciente e o manejo multidisciplinar, este documento visa melhorar a precisão diagnóstica, otimizar a seleção do tratamento e apoiar o atendimento seguro.

## INTRODUCTION

Hepatocellular carcinoma (HCC) is the most common primary malignant tumor of the liver and ranks among the leading causes of cancer-related death worldwide^5^. HCC typically arises in the setting of chronic liver disease, most often cirrhosis^79^. Its incidence continues to increase, particularly in regions with a high prevalence of chronic liver disease, including hepatitis B and C, alcoholic liver disease, and metabolic dysfunction-associated steatotic liver disease (MASLD)^48^. In Brazil, the distribution of risk factors reflects marked geographic heterogeneity. Viral hepatitis remains a major contributor to HCC in regions with higher endemicity, particularly in the North, whereas MASLD, alcohol-related liver disease, and cirrhosis associated with aging account for an increasing proportion of cases in more urbanized and industrialized areas. Together, these patterns underscore the multifactorial and regionally variable burden of HCC across the country^23^ ^,^ ^80^.

This clinical context makes patients with HCC particularly complex, as both tumor-related and liver-related factors simultaneously influence prognosis and therapeutic feasibility^14^. In many cases, liver function, portal hypertension, and overall clinical status play a more decisive role than tumor burden alone in determining eligibility for curative and non-curative treatments, reinforcing the need for a multidisciplinary and multimodal management approach.

Assessment of hepatic function is therefore central to clinical decision-making in HCC. Accurate evaluation of functional liver reserve directly influences treatment selection, surgical planning, and anticipated outcomes. This is particularly relevant for patients considered for liver resection, in whom preserved hepatic function is a prerequisite and major hepatectomy is generally restricted to those with well-compensated disease. Post-hepatectomy liver failure remains one of the leading causes of perioperative mortality, particularly when hepatic reserve is overestimated^57^ ^,^ ^77^.

Beyond defining liver-related risk, a detailed evaluation of tumor burden and disease stage is essential to inform management. Early and accurate diagnosis directly influences prognosis and treatment options, as therapeutic strategies are determined by both tumor stage and liver function at presentation^33^ ^,^ ^77^ ^,^ ^89^. The integration of tumor characteristics, hepatic reserve, portal hypertension, and clinical performance is formalized through staging systems, most notably the Barcelona Clinic Liver Cancer (BCLC) classification^60^. Although widely adopted, substantial heterogeneity exists within individual stages, particularly among patients with intermediate and advanced disease, highlighting the need for individualized interpretation within a multidisciplinary framework.

While laboratory markers such as alpha-fetoprotein (AFP) may support the diagnostic process, imaging plays a central role in the detection, characterization, and staging of HCC^19^. In patients with cirrhosis, chronic hepatitis B infection, or a history of HCC, noninvasive imaging criteria often provide sufficient diagnostic specificity, obviating the need for histopathological confirmation^47^. Nevertheless, biopsy remains necessary in selected situations, and inappropriate indication may expose patients to bleeding, tumor seeding, or delays in treatment. Knowing when to perform a biopsy-and, equally important, when to avoid it-is therefore a critical component of HCC evaluation.

This document, part of the Brazilian Multidisciplinary Consensus on Hepatocellular Carcinoma, addresses the comprehensive assessment and management of patients at the time of HCC diagnosis, including radiological evaluation, indications for biopsy, histopathological classification, clinical and functional liver assessment, evaluation of portal hypertension, diagnostic work-up, and staging. Through a multidisciplinary and evidence-based approach, the consensus aims to support standardized diagnostic practices and informed clinical decision-making.

## METHODS

This publication is part of a series of multidisciplinary consensus statements on HCC coordinated by the Brazilian Society of Surgical Oncology in collaboration with 13 other national medical societies. The following societies formally contributed to the consensus process: Brazilian Society of Clinical Oncology, Brazilian College of Surgeons, Brazilian College of Hepato-Pancreato-Biliary Surgery, Brazilian College of Digestive Surgery, Brazilian Society of Interventional Radiology and Endovascular Surgery, Brazilian Society of Radiotherapy, Brazilian Association of Organ Transplantation, Brazilian College of Radiology and Diagnostic Imaging, Brazilian Society of Hepatology, Brazilian Society of Pathology, Brazilian Federation of Gastroenterology, and the National Academy of Palliative Care.

The methodology used for this consensus followed the same structure and process as previously described by Coimbra et al.^11^, which detailed the complete framework adopted across all consensus phases.

In summary, the current document was developed with the active participation of 43 experts representing surgical oncology, hepatology, clinical oncology, hepatopancreatobiliary surgery, radiology, interventional radiology, liver transplantation, pathology, gastroenterology, radiation oncology, and palliative care. The scientific steering committee predefined clinically relevant questions addressing radiological and histopathological diagnosis, clinical assessment, diagnostic work-up, management of patients at diagnosis, and staging of HCC. These questions were discussed and refined through multidisciplinary meetings until they reached final recommendations. The grade of recommendation was classified as strong or weak, and the quality of supporting evidence was graded as high, moderate, or low. Strength of recommendations reflects both the quality of evidence and the balance between benefits, risks, feasibility, and resource availability.

## RESULTS

A total of 43 Brazilian experts from multiple specialties participated in this phase of the national consensus on HCC. The panel formulated 18 recommendations addressing key aspects of radiological and histopathological diagnosis, as well as the clinical assessment and management of patients with HCC. These recommendations aim to support clinical decision-making through standardized diagnostic criteria and reporting practices. The final recommendations are presented in Table 1.

**Table 1.** List of recommendations alongside the level of evidence and recommendation grades.

Recommendation		Evidence level	Recommendation grade
1	Standardized reporting using the LI-RADS^®^ should be adopted for contrast-enhanced CT and MRI in patients at risk for HCC, as it provides consistent lesion classification and facilitates communication and management decisions within multidisciplinary teams.	High	Strong
2	In high-risk patients, HCC may be diagnosed non-invasively when the LR-5 criteria are met. These include a lesion ≥10 mm with arterial phase hyperenhancement and washout appearance in the portal venous phase, with or without the presence of an enhancing capsule, or arterial phase hyperenhancement associated with threshold growth (≥50% increase in size within six months).	High	Strong
3	Patients with LR-3 observations should undergo follow-up with contrast-enhanced triphasic imaging at ≤6-month intervals to assess stability or progression. If the finding remains stable for approximately 18 months, the patient may return to routine ultrasound surveillance.	High	Strong
4	Management of LR-4 observations should be individualized and discussed within a multidisciplinary team. In clinical scenarios where a definitive diagnosis is required to guide management, such as in liver transplant candidates, liver biopsy is recommended. In patients with lower clinical suspicion, short-interval follow-up with contrast-enhanced triphasic imaging within ≤3 months may be considered to assess lesion progression.	High	Strong
5	MRI with hepatobiliary contrast may be considered as a complementary diagnostic tool in patients without decompensated cirrhosis and without significant cholestasis (total bilirubin <3 mg/dL) when additional lesion characterization is required. This includes differentiation of HCC from benign hepatocellular lesions, regenerative nodules, or dysplastic nodules, as well as cases of diagnostic uncertainty after multiphasic CT or extracellular contrast-enhanced MRI.	Low	Weak
6	In at-risk patients, biopsy should be considered for diagnostic confirmation of focal liver lesions ≥1 cm classified as LR-M, LR-4, or selected LR-3 after multidisciplinary evaluation when imaging is inconclusive and a definitive diagnosis is expected to alter clinical management. The choice of biopsy approach should be guided by lesion characteristics, patient anatomy, and institutional expertise.	Low	Weak
7	The 2019 WHO Classification of Tumors (and future updates) should be used in histopathological reports of HCC.	Moderate	Weak
8	Immunohistochemical confirmation of hepatocellular differentiation in carcinoma should be based primarily on arginase and HepPar-1, with preference for arginase due to its higher sensitivity and specificity. In poorly differentiated tumors with negative results for these markers, polyclonal CEA (with canalicular staining) and cytoplasmic TTF-1 should be used as ancillary tools. CD10 and AFP may be applied as complementary markers, although their diagnostic value is limited. In cases where distinction between HCC and benign hepatocytic lesions is required-such as regenerative or dysplastic nodules and hepatocellular adenomas-GPC-3, HSP-70, and GS should be evaluated. A diagnosis of HCC may be supported when at least two of these three markers are positive. Interpretation of GS must be cautious in lesions suspected of β-catenin-activated hepatocellular adenoma and should always be correlated with morphological features.	Moderate	Weak
9	For the diagnosis of combined hepatocellular-cholangiocarcinoma, morphological features of both hepatocellular and biliary differentiation must be clearly identified, either in distinct areas or intimately mixed. There is currently no minimum proportion required for either component. Immunohistochemical markers for hepatocellular and biliary differentiation, including CK19 and CK7, should be used to support the diagnosis.	Moderate	Weak
10	For incisional biopsy (needle or surgical), the minimum standardization for an HCC pathology report should include classification of the neoplasm (conventional or special subtype); description of the non-neoplastic parenchyma, including underlying liver disease and fibrosis grade, particularly cirrhosis status; percentage of viable tumor tissue (and necrosis) for potential molecular pathology studies; and presence or absence of vascular invasion. Although a negative finding has limited value, vascular invasion can often be detected even in needle biopsies. For resection specimens (nodulectomy, partial, or total hepatectomy), the report should include all items recommended for incisional biopsy, as well as: number, size, and location of neoplastic nodules; characterization of the vascular invasion, particularly the caliber of the involved vessels (capillaries, venules, small, medium, and large muscularized veins); local tumor extent (confined to the liver, capsule invasion, hilar soft tissues, or adjacent organs); response to previous therapy (systemic or ablative); surgical margins, including distance from the margins; and lymph node or distant metastases with histological documentation.	Low	Strong
11	For the initial clinical evaluation and staging of HCC, the work-up should include multiphasic contrast-enhanced abdominal CT or MRI, chest CT, serum alpha-fetoprotein measurement, viral hepatitis screening (HBsAg, anti-HBc IgG, anti-HBs, and anti-HCV), and laboratory assessment of hepatic function, a complete blood count with platelet count, and renal function. Upper gastrointestinal endoscopy should be performed in patients with cirrhosis or suspected clinically significant portal hypertension to evaluate for esophageal varices. Clinical decision-making should incorporate validated scoring systems, including the Child-Pugh score, MELD, ALBI, and ECOG performance status.	High	Strong
12	Alternative methods for hepatic function assessment should be considered selectively in patients with HCC. Liver elastography may be used to support fibrosis staging or confirm cirrhosis when standard clinical, laboratory, and imaging assessments are inconclusive. Liver biopsy may be indicated in selected cases to obtain a definitive assessment of fibrosis. Technetium-99m-mebrofenin hepatobiliary scintigraphy with SPECT/CT and gadoxetic acid-enhanced MRI are not established as hepatic function assessment tools.	Low	Weak
13	The BCLC system should be used for staging and prognostic prediction in patients with HCC; however, treatment decisions should not rely exclusively on BCLC stage and should be individualized based on multidisciplinary evaluation in centers with surgical and transplant expertise.	Moderate	Strong
14	Bone scintigraphy should not be performed routinely for staging in patients with HCC and should be reserved for cases with clinical symptoms or imaging findings suggestive of bone metastases.	Moderate	Weak
15	Routine PET-CT should not be performed for staging in patients with HCC. PET-CT may be considered in selected cases, particularly when there is suspected aggressive tumor biology or equivocal findings on conventional imaging, concern for extrahepatic disease, and the result is expected to influence clinical management.	High	Weak
16	A multidisciplinary approach should be adopted in the management of patients with HCC to support individualized treatment planning and optimize clinical outcomes.	Moderate	Strong
17	In patients with HBV-related HCC, antiviral therapy with potent nucleotide analogues should be initiated to reduce the risk of viral reactivation, decrease HCC recurrence, and improve overall survival, irrespective of the oncologic treatment strategy.	Moderate	Strong
18	In patients with HCV-related HCC, DAA therapy should be offered after a complete response to curative-intent treatments, including surgical resection or local ablation. In candidates for liver transplantation, the timing of DAA therapy should be individualized, considering regional waiting times, availability of HCV-positive donor organs, and the severity of underlying liver dysfunction. In patients who are not candidates for curative treatment, routine DAA therapy is not recommended, and antiviral treatment should be individualized based on treatment costs, tumor burden, expected survival, liver function, and patient preferences. This recommendation reflects current evidence on competing risks rather than a lack of antiviral efficacy.	Low	Weak

LI-RADS: Liver Imaging Reporting and Data System; LR: LI-RADS; HCC: hepatocellular carcinoma; PET-CT: positron emission tomography-computed tomography; HBV: hepatitis B virus; DAA: Direct-Acting Antiviral therapy; MRI: magnetic resonance imaging; CT: computed tomography; MELD: Model for End-Stage Liver Disease; BCLC: Barcelona Clinic Liver Cancer; SPECT/CT: single-photon emission computed tomography/computed tomography; ALBI: Albumin-Bilirubin; ECOG: Eastern Cooperative Oncology Group; WHO: World Health Organization; CK: cytokeratin; HSP-70: heat shock protein 70; CEA: carcinoembryonic antigen; AFP: alpha-fetoprotein; TTF-1: thyroid transcription factor; HepPar-1: hepatocyte paraffin 1; CD: cluster of differentiation.

### What is the importance of standardizing radiological reports according to the Liver Imaging Reporting and Data System?

In patients at high risk for HCC, non-invasive imaging criteria may allow diagnosis with high specificity without the need for histopathological confirmation if typical radiological features are present on contrast-enhanced imaging, such as multiphasic computed tomography (CT) or magnetic resonance imaging (MRI)^19^ ^,^ ^37^ ^,^ ^47^ ^,^ ^82^ ^,^ ^95^. To improve diagnostic consistency, the American College of Radiology developed the LI-RADS^®^ (Table 2). For patients at high risk of HCC, the LI-RADS algorithm incorporates major imaging features characteristic of HCC, along with ancillary features, to assign a final category reflecting the probability of malignancy based on contrast-enhanced CT or MRI examinations^1^ ^,^ ^9^. A LI-RADS category applies to individual focal liver observations rather than to the overall examination. On contrast-enhanced MRI and CT, LI-RADS v2018 major imaging features for HCC are: non-rim arterial phase hyperenhancement; non-peripheral washout appearance in the portal or delayed phase (for hepatobiliary contrast agents, this applies only to the portal phase); presence of an enhancing capsule, visualized as a smooth and uniform rim in portal, delayed, or transitional phases; lesion size, defined as the maximum outer-to-outer measurement; and threshold growth.

**Table2.** Summary of computed tomography and magnetic resonance imaging diagnostic Liver Imaging Reporting and Data System (LI-RADS) categories for hepatocellular carcinoma in at-risk patients.

	Conceptual definition	CT/MRI diagnostic criteria (LI-RADS)
LR-NC (Not categorizable)	Observation that cannot be meaningfully categorized because image omission or degradation prevents assessment of one or more major features.	Both of the following: (1) One or more major features cannot be assessed because of image omission or degradation; and (2) as a direct result, the possible categories range from those in which cancer is unlikely (LR-1 or LR-2) to those in which cancer is likely (LR-4, LR-5, or LR-M).
LR-1 (Definitely benign)	100% certainty that observation is nonmalignant.	Classic benign lesions.
LR-2 (Probably benign)	High probability but not 100% certainty that observation is nonmalignant.	Criteria for an LR-2 distinctive nodule: size <20 mm, no major features, no LR-M features, and no ancillary features favoring malignancy.
LR-3 (Intermediate probability of malignancy)	Nonmalignant and malignant entities each have moderate probability.	Either (A) Nonrim arterial phase hyperenhancement and size <20 mm with no additional major features; or (B) Arterial phase hypo- or isoenhancement and either size <20 mm with ≤1 additional major feature, or size ≥20 mm with no additional major features.
LR-4 (Probably HCC)	High probability but not 100% certainty that observation is HCC.	Either (A) Nonrim arterial phase hyperenhancement and one of the following: size <10 mm with ≥1 additional major feature; or size 10-19 mm with “capsule” and no other major features; or size ≥20 mm with no additional major feature; or (B) Arterial phase hypo- or isoenhancement and one of the following: size <20 mm with ≥2 additional major features; or size ≥20 mm with ≥1 additional major feature.
LR-5 (Definitely HCC)	100% certainty observation is HCC.	Nonrim arterial phase hyperenhancement and one of the following: size 10-19 mm with nonperipheral “washout” and no other major features; or size 10-19 mm with ≥50% size increase in ≤6 months and no other major features; or size ≥20 mm with ≥1 additional major feature.
LR-TIV (Tumor in vein)	100% certainty there is malignancy with tumor in vein.	Presence of definite enhancing soft tissue in a vein, regardless of visualization of a parenchymal mass.
LR-M (Probably or definitely malignant, not specific for HCC)	High probability or 100% certainty observation is malignant but features are not specific for HCC. Note: LR-M does not exclude HCC; it indicates a substantial probability of a malignant neoplasm other than HCC.	Either (A) Targetoid mass with one or more of the following: rim arterial phase hyperenhancement; peripheral washout appearance; delayed central enhancement; targetoid diffusion restriction; targetoid transitional phase or hepatobiliary phase signal intensity; or (B) Nontargetoid mass that does not meet LR-5 criteria and has no tumor in vein, with ≥1 of the following: infiltrative appearance; marked diffusion restriction; necrosis or severe ischemia; other feature suggesting non-HCC malignancy.

CT/MRI: computed tomography/magnetic resonance; LI-RADS: Liver Imaging Reporting and Data System; LR: LI-RADS; HCC: hepatocellular carcinoma.

Source: adapted from Chernyak et al.^9^.

The application of LI-RADS (LR) follows a flowchart algorithm, beginning with the exclusion of observations that cannot be reliably assessed (LR-NC). It then evaluates the presence or absence of tumor in the vein (LR-TIV), as well as the presence of definitely benign lesions (LR-1), probably benign lesions (LR-2), and observations with high suspicion for malignancy but without typical features of HCC (LR-M). Following these initial steps, a diagnostic table categorizes solid observations into LR-3 (intermediate probability), LR-4 (probably HCC), or LR-5 (definitely HCC) based on the presence of major imaging features. Ancillary features may be used to adjust the final categorization upward or downward but cannot upgrade an LR-4 observation to an LR-5 due to their limited specificity for HCC^1^ ^,^ ^9^.

The use of LI-RADS provides at least three major benefits: it standardizes nomenclature and diagnostic criteria, improves communication among the multidisciplinary teams involved in the care of high-risk patients or those with HCC, and facilitates data collection and collaboration in the research setting. The application of the LI-RADS algorithm results in high specificity (>90%) and positive predictive value (>95%) for HCC in lesions categorized as LR-5, while maintaining high sensitivity for malignancy even in non-HCC-specific categories such as LR-4 and LR-M. The algorithm also informs individualized clinical decision-making through structured management recommendations for each diagnostic category^13^ ^,^ ^20^ ^,^ ^39^ ^,^ ^78^.

### Recommendation 1

Standardized reporting using the LI-RADS^®^ should be adopted for contrast-enhanced CT and MRI in patients at risk for HCC, as it provides consistent lesion classification and facilitates communication and management decisions within multidisciplinary teams.

Evidence level: High

Recommendation grade: Strong

### What are the currently accepted criteria for the radiological diagnosis of HCC?

Imaging-based diagnosis using multiphasic CT or MRI with contrast is indicated in patients with high clinical suspicion, such as those with a hepatic nodule detected on ultrasound and elevated AFP, as well as in patients with incidental hepatic lesions^47^ ^,^ ^49^. Several studies suggest that arterial-phase hyperenhancement on a single imaging modality is sufficient to support the diagnosis of HCC in cirrhotic patients with 1-2 cm nodules detected during surveillance, potentially reducing the need for biopsy^49^ ^,^ ^60^.

In patients at high risk for HCC, non-invasive imaging criteria enable diagnosis with high specificity when typical radiologic hallmarks are present. In these cases, histopathological confirmation is usually not necessary^19^ ^,^ ^37^ ^,^ ^47^ ^,^ ^84^ ^,^ ^95^. Lee et al.^39^ conducted a systematic review demonstrating that the CT or MRI LR-5 category has a pooled specificity of 92% (95% [confidence interval] CI: 88-95%) for diagnosing HCC.

### Recommendation 2

In high-risk patients, HCC may be diagnosed non-invasively when the LR-5 criteria are met. These include a lesion ≥10 mm with arterial phase hyperenhancement and washout appearance in the portal venous phase, with or without the presence of an enhancing capsule, or arterial phase hyperenhancement associated with threshold growth (≥50% increase in size within six months).

Evidence level: High

Recommendation grade: Strong

### What should be the approach or management of LR-3 lesions?

Patients with LR-3 observations are at a clinically meaningful risk of developing HCC, although the magnitude and timing of this risk are heterogeneous. A systematic review by Kanneganti et al.^35^ evaluating patients with cirrhosis and LR-3 observations reported incident HCC rates of 1.2 to 12.5% at 12 months and 4.2 to 44.4% with longer follow-up, while an additional proportion progressed to higher-risk imaging categories. These findings support the need for close radiological monitoring, as the risk of malignant transformation increases with continued observation. Accordingly, patients with LR-3 lesions should undergo follow-up with contrast-enhanced multiphasic CT or MRI at intervals of six months or less to assess lesion stability or progression. Returning to ultrasound-based surveillance is not recommended in this setting.

### Recommendation 3

Patients with LR-3 observations should undergo follow-up with contrast-enhanced triphasic imaging at ≤6-month intervals to assess stability or progression. If the finding remains stable for approximately 18 months, the patient may return to routine ultrasound surveillance.

Evidence level: High

Recommendation grade: Strong

### What should be the approach or management of LR-4 lesions?

LR-4 observations are associated with a high risk of HCC. Kanneganti et al.^35^ reported incident HCC rates ranging from 30.8 to 44.0% at 12 months and from 30.9 to 71.0% with longer follow-up. Conversely, 6 to 42% of LR-4 observations were downgraded to LR-3 or lower during follow-up, indicating heterogeneity in lesion behavior. A large individual participant data meta-analysis evaluating CT, MRI, and LI-RADS further demonstrated a pooled positive predictive value of approximately 80.8% (95%CI 71.0-87.9) for HCC among LR-4 observations, confirming that most lesions in this category represent probable HCC^65^. Importantly, the study showed that most combinations of major imaging features within LR-4 had similar diagnostic performance, supporting the validity of the current categorization, although certain feature patterns-particularly those lacking arterial phase hyperenhancement-were associated with lower predictive value^1^. These data support an approach based on multidisciplinary evaluation and timely diagnostic clarification.

### Recommendation 4

Management of LR-4 observations should be individualized and discussed within a multidisciplinary team. In clinical scenarios where a definitive diagnosis is required to guide management, such as in liver transplant candidates, liver biopsy is recommended. In patients with lower clinical suspicion, short-interval follow-up with contrast-enhanced triphasic imaging within ≤3 months may be considered to assess lesion progression.

Evidence level: High

Recommendation grade: Strong

### When should an MRI with hepatobiliary contrast be indicated for HCC?

Hepatobiliary contrast agents are taken up by functional hepatocytes and excreted via the biliary system, thereby allowing assessment of hepatocellular function. In practical terms, this property helps distinguish hepatocellular lesions with preserved hepatocyte function, such as focal nodular hyperplasia, which typically demonstrate iso- or hyperintensity in the hepatobiliary phase, from lesions with impaired or absent hepatocyte function, such as most HCC, which most often appear hypointense. In cirrhotic livers, this mechanism also allows differentiation between regenerative nodules and HCC. Regenerative nodules and low-grade dysplastic nodules preserve hepatocellular function and typically demonstrate hepatobiliary contrast uptake similar to that of the surrounding liver parenchyma. High-grade dysplastic nodules, although characterized by progressive loss of portal supply and development of abnormal arterialization, may still contain functional hepatocytes and therefore often retain hepatobiliary contrast, appearing iso- or hyperintense in the hepatobiliary phase. In contrast, the uptake of hepatobiliary contrast by HCC depends on tumor differentiation. Well-differentiated HCCs may retain contrast due to residual hepatocyte function, whereas poorly differentiated or undifferentiated HCCs lack functional hepatocytes and consistently appear hypointense on hepatobiliary phase imaging^22^.

MRI with hepatobiliary contrast for detecting lesions ≤2 cm has shown acceptable accuracy when criteria are applied in the appropriate clinical context in patients with known liver disease^21^ ^,^ ^49^. Within the LI-RADS framework, findings in the hepatobiliary phase should be interpreted as ancillary features that may refine diagnostic confidence but do not replace major imaging criteria for HCC. However, in patients with decompensated cirrhosis or significant cholestasis, hepatobiliary contrast uptake is reduced due to global impaired hepatocyte function and disrupted biliary excretion, resulting in poor hepatobiliary phase lesion-to-liver contrast, which often reduces diagnostic accuracy. Elevated total bilirubin levels (typically >3 mg/dL) are associated with suboptimal image quality and limited clinical utility of hepatobiliary contrast agents, and therefore, in that setting, extracellular contrast agents should be preferred^34^.

### Recommendation 5

MRI with hepatobiliary contrast may be considered as a complementary diagnostic tool in patients without decompensated cirrhosis and without significant cholestasis (total bilirubin <3 mg/dL) when additional lesion characterization is required. This includes differentiation of HCC from benign hepatocellular lesions, regenerative nodules, or dysplastic nodules, as well as cases of diagnostic uncertainty after multiphasic CT or extracellular contrast-enhanced MRI.

Evidence level: Low

Recommendation grade: Weak

### When is a biopsy indicated for a focal liver lesion, and which approach should be preferred (percutaneous, ultrasound-guided, or CT-guided)?

In contemporary series, the risk of tumor seeding along the biopsy tract is low-generally <1%-but clinically significant complications such as bleeding and false-negative results must also be considered^52^. Moreover, in patients at risk for HCC, the diagnosis can often be established noninvasively using dynamic imaging, thereby limiting the need for tissue confirmation. Therefore, biopsy is reserved for situations in which imaging is inconclusive or when histological confirmation is expected to directly influence clinical management.

It is important to distinguish between a biopsy performed for diagnostic confirmation and one performed for tissue characterization. Diagnostic biopsy aims to establish the presence and histological subtype of malignancy when imaging findings are insufficient for a definitive diagnosis. Tissue samples may also allow immunohistochemical or molecular analyses relevant to prognosis or clinical trial eligibility; however, such analyses are not routinely required for standard management of HCC and should not constitute the sole indication for biopsy.

A liver biopsy may be indicated when the diagnosis of HCC cannot be established by dynamic imaging (triphasic CT or MRI) in hepatic lesions ≥1 cm in high-risk patients^10^ ^,^ ^47^ ^,^ ^56^. Biopsy is particularly relevant when imaging findings are non-specific for HCC, including LR-4 or LR-M observations. After multidisciplinary discussion, biopsy should be considered in clinical scenarios requiring a high positive predictive value, when histological confirmation is expected to directly alter management, such as decisions regarding surgical resection, liver transplantation, or systemic therapy^49^.

The decision between ultrasound- and CT-guided biopsy should be based on lesion characteristics, patient-specific factors, and local expertise. In patients with advanced cirrhosis, ultrasound may have limited sensitivity for detecting focal lesions, making CT guidance preferable in selected cases^54^.

### Recommendation 6

In at-risk patients, biopsy should be considered for diagnostic confirmation of focal liver lesions ≥1 cm classified as LR-M, LR-4, or selected LR-3 after multidisciplinary evaluation when imaging is inconclusive and a definitive diagnosis is expected to alter clinical management. The choice of biopsy approach should be guided by lesion characteristics, patient anatomy, and institutional expertise.

Evidence level: Low

Recommendation grade: Weak

### Which histopathological classification should be used for HCC?

**Figure 1.**
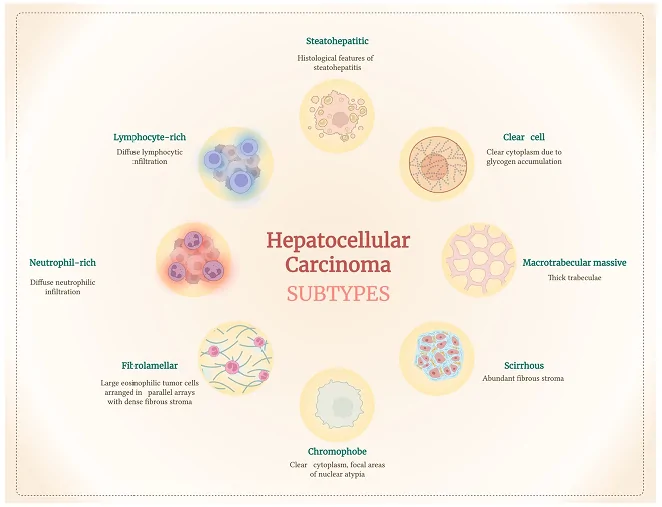
The hepatocellular carcinoma histopathological subtypes according to the World Health Organization Classification of Tumors of the Digestive System (5th edition).

HCC is no longer considered a single tumor entity but rather a heterogeneous group of neoplasms with distinct clinical, morphological, and molecular features. This heterogeneity is reflected in the 2019 World Health Organization (WHO) Classification of Tumors of the Digestive System (5th edition), which is primarily histopathology-based and provides standardized morphological criteria for HCC variants, while increasingly incorporating supportive molecular correlates^36^ ^,^ ^46^. The WHO recognizes eight special subtypes in addition to conventional HCC (Figure 1): steatohepatitic, clear cell, macrotrabecular-massive, scirrhous, chromophobe, fibrolamellar, neutrophil-rich, and lymphocyte-rich. The clinical importance of this framework lies in standardizing reporting and enabling clinically meaningful risk stratification, as some variants are associated with distinct outcomes and clinicopathologic profiles^74^. For example, macrotrabecular-massive HCC is associated with poorer prognosis. In contrast, lymphocyte-rich HCC tends to have a more favorable outcome and is reported to exhibit frequent PD-L1 expression. Steatohepatitic HCC is strongly associated with metabolic syndrome and is more frequently found in non-cirrhotic livers^20^ ^,^ ^36^ ^,^ ^43^. Beyond morphology, molecular subtyping is emerging as a complementary layer with potential predictive implications in advanced disease; for instance, multi-omic lineage-related molecular subtypes have shown differential response to atezolizumab plus bevacizumab, supporting the broader move toward biologically informed personalization of therapy^40^ ^,^ ^43^. As systemic therapies evolve, accurate histological classification and selective use of biopsy can support more precise tumor characterization, including when tissue is needed for molecular and immunohistochemical profiling^68^.

### Recommendation 7

The 2019 WHO Classification of Tumors (and future updates) should be used in histopathological reports of HCC.

Evidence level: Moderate

Recommendation grade: Weak

### What immunohistochemical markers should be used for HCC definition?

Immunohistochemistry plays a central role in the diagnosis of HCC by confirming hepatocellular differentiation, supporting the distinction between benign and malignant hepatocytic lesions, aiding in the recognition of WHO-defined histopathological subtypes, and contributing to prognostic stratification. At present, its role is primarily diagnostic and classificatory, and it does not have a validated function in selecting systemic therapies for HCC^40^.

For characterizing hepatocellular differentiation in carcinoma and distinguishing HCC from other tumors, HepPar-1 (hepatocyte paraffin 1) and arginase are the two primary immunohistochemical markers, with arginase demonstrating greater sensitivity and specificity^51^. In poorly differentiated tumors in which these markers are negative, polyclonal carcinoembryonic antigen (CEA) can be used because of its bile canalicular staining pattern; however, its specificity is lower, and interpretation depends on the pathologist’s experience^83^. Cytoplasmic (non-nuclear) thyroid transcription factor-1 (TTF-1) expression is also considered a marker of hepatocellular differentiation and may be particularly useful in poorly differentiated cases^2^. Other markers, such as cluster of differentiation 10 (CD10) and AFP, may be applied, but generally have limited diagnostic utility^41^ ^,^ ^51^. For the assessment of malignancy-especially when distinguishing HCC from benign hepatocytic lesions such as regenerative or dysplastic nodules in cirrhotic livers, or hepatocellular adenomas in non-cirrhotic livers-the most widely used markers are glypican-3 (GPC-3), heat shock protein (HSP-70), and glutamine synthetase (GS). When the malignant potential of a hepatic nodule is uncertain, positivity for at least two of these three markers is considered sufficient to support a diagnosis of HCC. However, GS may also be expressed in hepatocellular adenomas with β-catenin mutations, requiring careful correlation with morphological findings^17^ ^,^ ^51^ ^,^ ^63^.

### Recommendation 8

Immunohistochemical confirmation of hepatocellular differentiation in carcinoma should be based primarily on arginase and HepPar-1, with preference for arginase due to its higher sensitivity and specificity. In poorly differentiated tumors with negative results for these markers, polyclonal CEA (with canalicular staining) and cytoplasmic TTF-1 should be used as ancillary tools. CD10 and AFP may be applied as complementary markers, although their diagnostic value is limited. In cases where distinction between HCC and benign hepatocytic lesions is required-such as regenerative or dysplastic nodules and hepatocellular adenomas-GPC-3, HSP-70, and GS should be evaluated. A diagnosis of HCC may be supported when at least two of these three markers are positive. Interpretation of GS must be cautious in lesions suspected of β-catenin-activated hepatocellular adenoma and should always be correlated with morphological features.

Evidence level: Moderate

Recommendation grade: Weak

### What are the histopathological and immunohistochemical criteria for diagnosing combined hepatocellular-cholangiocarcinoma?

Combined hepatocellular-cholangiocarcinoma, previously referred to as hepatobiliary carcinoma, is a primary liver malignancy that displays unequivocal features of both hepatocellular and biliary differentiation. According to current histopathological standards, the diagnosis requires clear identification of the morphological components of both HCC and cholangiocarcinoma, which may occur in distinct regions or in an intimately mixed pattern. No minimum proportion of either element is currently required to establish the diagnosis^69^ ^,^ ^70^ ^,^ ^91^. Morphological assessment remains the diagnostic cornerstone, but immunohistochemistry can provide supportive evidence. Markers of hepatocellular differentiation, such as those described previously, should be used in combination with markers of biliary differentiation, particularly cytokeratin (CK) 19 and CK7.

### Recommendation 9

For the diagnosis of combined hepatocellular-cholangiocarcinoma, morphological features of both hepatocellular and biliary differentiation must be clearly identified, either in distinct areas or intimately mixed. There is currently no minimum proportion required for either component. Immunohistochemical markers for hepatocellular and biliary differentiation, including CK19 and CK7, should be used to support the diagnosis.

Evidence level: Moderate

Recommendation grade: Weak

### What are the requirements for standardizing histopathological reports in HCC?

Standardization of pathology reports in HCC is essential to ensure consistency in diagnosis, enable prognostic assessment, and guide therapeutic decisions. Comprehensive histological evaluation provides critical data not only on tumor classification and subtype, but also on background liver disease, tumor viability, vascular invasion, and treatment response. In this context, minimum reporting standards improve multidisciplinary communication and allow for the integration of histopathological findings into structured clinical management algorithms.

### Recommendation 10

For incisional biopsy (needle or surgical), the minimum standardization for an HCC pathology report should include classification of the neoplasm (conventional or special subtype); description of the non-neoplastic parenchyma, including underlying liver disease and fibrosis grade, particularly cirrhosis status; percentage of viable tumor tissue (and necrosis) for potential molecular pathology studies; and presence or absence of vascular invasion. Although a negative finding has limited value, vascular invasion can often be detected even in needle biopsies. For resection specimens (nodulectomy, partial, or total hepatectomy), the report should include all items recommended for incisional biopsy, as well as: number, size, and location of neoplastic nodules; characterization of the vascular invasion, particularly the caliber of the involved vessels (capillaries, venules, small, medium, and large muscularized veins); local tumor extent (confined to the liver, capsule invasion, hilar soft tissues, or adjacent organs); response to previous therapy (systemic or ablative); surgical margins, including distance from the margins; and lymph node or distant metastases with histological documentation.

Evidence level: Low

Recommendation grade: Strong

### What is the recommended initial diagnostic work-up, including the required tests and clinical evaluation, for the management of HCC?

Clinical evaluation and hepatic function assessment are central to HCC management, as prognosis and treatment feasibility are determined not only by tumor burden but also by the severity of underlying liver disease and patient functional status. Multiple observational and surgical series have consistently demonstrated that baseline hepatic reserve and clinical performance are independently associated with survival and treatment-related morbidity across curative and non-curative modalities, including liver resection, transplantation, locoregional therapies, and systemic treatment^12^ ^,^ ^24^ ^,^ ^45^ ^,^ ^61^ ^,^ ^85^. Accurate characterization of intrahepatic tumor extent and vascular involvement is essential for determining resectability, transplant eligibility, and suitability for locoregional therapies. Thoracic evaluation helps identify extrahepatic disease. Serum AFP, although imperfect, has been widely used as a biomarker in HCC and has been associated with tumor burden, biological aggressiveness, and survival, supporting its role in baseline assessment and prognostic stratification. Determination of the etiology of chronic liver disease, particularly viral hepatitis, is also clinically relevant, as it influences prognosis and long-term management strategies.

Laboratory evaluation of hepatic function provides insight into synthetic capacity, hepatocellular injury, and cholestasis, which are critical determinants of treatment tolerance and postoperative risk. Platelet count serves as an indirect marker of portal hypertension, while renal function has been shown to influence prognosis and eligibility for both surgical and systemic therapies. Identification of esophageal varices through upper gastrointestinal endoscopy offers additional information on the presence and severity of portal hypertension^25^ ^,^ ^28^ ^,^ ^33^.

Clinical decision-making in HCC commonly integrates composite scoring systems that combine hepatic function and patient performance. The Child-Pugh score, Model for End-Stage Liver Disease (MELD) score, Albumin-Bilirubin (ALBI) score, and Eastern Cooperative Oncology Group (ECOG) performance status have all demonstrated prognostic value and have been correlated with survival, treatment tolerance, and response across different therapeutic settings^33^ ^,^ ^55^ ^,^ ^75^.

Assessment of portal hypertension is particularly relevant in HCC because it reflects the severity of the chronic liver disease and directly influences surgical risk and long-term outcomes. Although direct measurement of the hepatic venous pressure gradient remains the reference standard, limited availability has led to reliance on noninvasive surrogates such as imaging findings, splenomegaly, thrombocytopenia, and esophageal varices, all of which have been associated with clinically significant portal hypertension^6^. Additional tools, including liver elastography, have been used to further characterize hepatic functional reserve and fibrosis severity, providing complementary information to clinical and laboratory assessment^7^.

### Recommendation 11

For the initial clinical evaluation and staging of HCC, the work-up should include multiphasic contrast-enhanced abdominal CT or MRI, chest CT, serum alpha-fetoprotein measurement, viral hepatitis screening (HBsAg, anti-HBc IgG, anti-HBs, and anti-HCV), and laboratory assessment of hepatic function, a complete blood count with platelet count, and renal function. Upper gastrointestinal endoscopy should be performed in patients with cirrhosis or suspected clinically significant portal hypertension to evaluate for esophageal varices. Clinical decision-making should incorporate validated scoring systems, including the Child-Pugh score, MELD, ALBI, and ECOG performance status.

Evidence level: High

Recommendation grade: Strong

### When should alternative methods for hepatic function assessment be used in patients with HCC?

Assessment of hepatic function using alternative methods may be relevant in HCC in two main clinical contexts: clarification of fibrosis or cirrhosis status when standard clinical, laboratory, and imaging findings are inconclusive; and evaluation of functional liver reserve for therapeutic planning.

Liver elastography, performed using ultrasound- or MRI-based techniques, has demonstrated high diagnostic accuracy for fibrosis detection and staging, with reported accuracy ranging from 85 to 90%, supporting its role as a noninvasive method for cirrhosis confirmation^4^ ^,^ ^31^ ^,^ ^93^. In situations in which noninvasive assessment is inconclusive, liver biopsy may still be required to confirm the diagnosis of cirrhosis.

Beyond risk stratification, evaluation of functional liver reserve is particularly relevant for treatment planning, especially in candidates for liver resection. Hepatobiliary scintigraphy using technetium-99m-mebrofenin combined with SPECT/CT allows quantitative assessment of hepatic function and provides information on functional distribution at the segmental or lobar level, which may be useful when preoperative evaluation of functional reserve is required^15^ ^,^ ^59^. Indocyanine green clearance testing offers an estimate of global hepatic excretory function. However, the absence of segmental or lobar functional assessment limits the accuracy of postoperative outcome prediction^15^ ^,^ ^16^ ^,^ ^59^.

### Recommendation 12

Alternative methods for hepatic function assessment should be considered selectively in patients with HCC. Liver elastography may be used to support fibrosis staging or confirm cirrhosis when standard clinical, laboratory, and imaging assessments are inconclusive. Liver biopsy may be indicated in selected cases to obtain a definitive assessment of fibrosis. Technetium-99m-mebrofenin hepatobiliary scintigraphy with SPECT/CT and gadoxetic acid-enhanced MRI are not established as hepatic function assessment tools.

Evidence level: Low

Recommendation grade: Weak

### Should the BCLC system be used to guide staging, prognostic prediction, and treatment decisions in patients with HCC?

**Figure 2.**
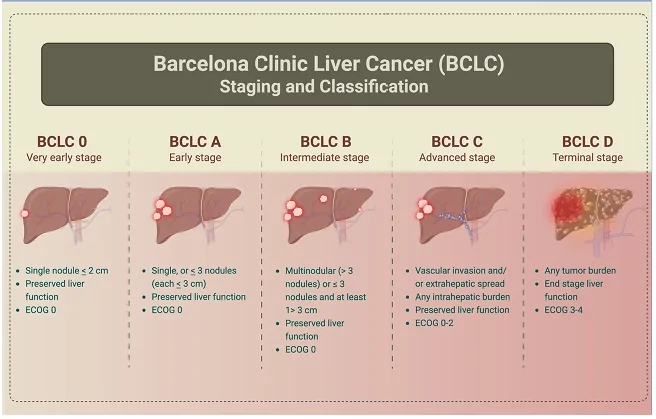
The Barcelona Clinic Liver Cancer staging and classification.

Staging in HCC is fundamental for prognostic stratification and therapeutic planning, and several staging systems have been proposed, including the American Joint Committee on Cancer (AJCC), Okuda Staging System (Okuda), Cancer of the Liver Italian Program (CLIP), and BCLC classifications. Among these, the BCLC system has become the most widely used framework for staging and prognostic prediction, mainly due to its integration of tumor burden, liver function, and clinical performance, as well as its reproducibility across different clinical settings^60^. See Figure 2.

Despite its broad adoption, the BCLC system has been consistently reported to have significant limitations. Substantial heterogeneity exists within intermediate-stage disease, particularly among stage B patients, in whom tumor size, number of nodules, and underlying liver function vary widely and are associated with different outcomes^29^ ^,^ ^30^ ^,^ ^87^. Similarly, stage C encompasses both locally advanced and metastatic disease, groups that differ markedly in prognosis and therapeutic options, complicating uniform treatment allocation^77^. In addition, the BCLC system does not fully incorporate tumor biology, therapeutic response, or the expanding indications for surgical resection and liver transplantation beyond the very early and early stages, which have been increasingly reported in clinical practice^81^.

Treatment decision-making in HCC has become progressively more complex with the development of new systemic therapies, refinements in locoregional treatments, and the broader application of surgical approaches. In this context, multidisciplinary evaluation has been associated with improved treatment selection and overall survival and has emerged as a critical component of contemporary HCC management^64^. These developments highlight the need to interpret staging systems within a broader clinical framework rather than as rigid treatment algorithms.

### Recommendation 13

The BCLC system should be used for staging and prognostic prediction in patients with HCC; however, treatment decisions should not rely exclusively on BCLC stage and should be individualized based on multidisciplinary evaluation in centers with surgical and transplant expertise.

Evidence level: Moderate

Recommendation grade: Strong

### Should bone scintigraphy be performed routinely for staging in patients with HCC?

Although bone metastases can occur in advanced HCC, their incidence at initial diagnosis is low, limiting the potential impact of systematic skeletal evaluation in unselected patients. Rodriguez-Alvarez et al.^62^ analyzed 238 patients staged using abdominal imaging, chest CT, and bone scintigraphy. Extrahepatic disease was present in 18% of patients, and 8% had positive bone scintigraphy results. However, among the positive cases, only four were true positives, and none affected staging or therapy plans.

Routine use of bone scintigraphy presents important limitations in clinical practice. The examination incurs additional costs and requires resources, with variable availability across healthcare systems. It also exposes patients to ionizing radiation and has a recognized risk of false-positive findings related to degenerative changes, trauma, or inflammatory bone disease, which may lead to unnecessary additional investigations, delayed treatment decisions, and increased patient burden without clear clinical benefit. Consequently, bone scintigraphy should not be performed routinely for HCC staging and should be reserved for selected patients, particularly those with bone-related symptoms or suspicious findings on CT or MRI^26^ ^,^ ^32^.

### Recommendation 14

Bone scintigraphy should not be performed routinely for staging in patients with HCC and should be reserved for cases with clinical symptoms or imaging findings suggestive of bone metastases.

Evidence level: Moderate

Recommendation grade: Weak

### What is the role of PET-CT in the staging of patients with HCC?

Prospective evidence from the positron emission tomography (PET)-HCC01 multicenter study provides important data on the clinical impact of PET-CT in patients with HCC. In this study, 215 patients with newly diagnosed HCC, staged as BCLC A-C, underwent standard morphological imaging followed by PET-CT with fluorine-18 fluorodeoxyglucose (18F-FDG) and 18F-fluorocholine. PET-CT identified potential new lesions in 19 patients (9%), but only six of these findings were confirmed as HCC on follow-up, including one adrenal lesion, two bone lesions, two lymph node lesions, and one intrahepatic lesion. PET-CT led to a change in BCLC stage in ten patients, reflecting reclassification across stages A, B, and C. Despite these staging changes, planned treatment was modified in only four patients (2% of the cohort), a proportion that was below the study’s prespecified threshold for clinical relevance^50^.

These findings underscore the limited incremental value of PET-CT for routine staging. One of the main reasons for this limited performance is the metabolic heterogeneity of HCC. Well-differentiated HCC, the most common histopathological pattern, closely resembles normal hepatocytes and exhibits metabolic behavior similar to that of normal hepatocytes, resulting in low tracer uptake and reduced sensitivity. Consequently, PET-CT shows limited accuracy in distinguishing intrahepatic HCC lesions from surrounding liver parenchyma^44^ ^,^ ^90^.

Conversely, PET-CT may provide additional information in selected contexts. 18F-FDG PET-CT has demonstrated utility in detecting extrahepatic disease and in identifying tumors with aggressive biological behavior. Increased metabolic activity and higher metabolic tumor volume have been associated with poorer prognosis and inferior treatment outcomes, suggesting a role for PET-derived parameters in prognostication. However, variability in sensitivity and specificity, together with cost, limited availability, and radiation exposure, restrict its broader clinical application^44^.

### Recommendation 15

Routine PET-CT should not be performed for staging in patients with HCC. PET-CT may be considered in selected cases, particularly when there is suspected aggressive tumor biology or equivocal findings on conventional imaging, concern for extrahepatic disease, and the result is expected to influence clinical management.

Evidence level: High

Recommendation grade: Weak

### Should the management of patients with HCC be guided by a multidisciplinary team approach?

Management of HCC requires complex clinical decision-making that integrates tumor burden, patient comorbidities, and the severity of underlying liver disease^71^. Multidisciplinary discussion enables the integration of perspectives from hepatology, surgery, oncology, radiology, interventional radiology, transplantation, and palliative care, thereby facilitating individualized treatment strategies tailored to both tumor characteristics and liver function^88^.

Observational studies have consistently shown that multidisciplinary team-based management of HCC is associated with improved adherence to evidence-based therapies, more appropriate treatment allocation, and improved overall survival when compared with non-coordinated care models^8^ ^,^ ^53^ ^,^ ^92^. These benefits appear to be driven by more accurate staging, improved patient selection for curative and non-curative therapies, and timely treatment sequencing.

### Recommendation 16

A multidisciplinary approach should be adopted in the management of patients with HCC to support individualized treatment planning and optimize clinical outcomes.

Evidence level: Moderate

Recommendation grade: Strong

### How should hepatitis B virus infection be managed in patients diagnosed with HCC?

Strong evidence indicates that antiviral therapy for hepatitis B virus (HBV) improves outcomes in patients with HCC. Suppression of viral replication reduces the risk of HBV reactivation, limits hepatic decompensation, and is associated with lower rates of HCC recurrence after curative-intent therapies, ultimately improving overall survival^42^ ^,^ ^73^. Nucleotide analogs, including entecavir, tenofovir disoproxil fumarate, and tenofovir alafenamide, are effective in achieving sustained viral suppression. A meta-regression analysis including 14 studies and 1,284 patients demonstrated that antiviral therapy significantly reduces HCC recurrence and improves overall survival in patients with HBV-related HCC^94^.

The importance of antiviral therapy is further underscored in patients receiving systemic treatment for HCC. Anti-angiogenic agents and immune checkpoint inhibitors may increase the risk of HBV reactivation, although HBV-positive patients have been underrepresented in pivotal clinical trials evaluating these therapies^18^ ^,^ ^38^ ^,^ ^86^. Available real-world data suggest that HBV reactivation can occur during immunotherapy, even among patients receiving antiviral prophylaxis, highlighting the need for systematic viral suppression and monitoring^72^.

### Recommendation 17

In patients with HBV-related HCC, antiviral therapy with potent nucleotide analogues should be initiated to reduce the risk of viral reactivation, decrease HCC recurrence, and improve overall survival, irrespective of the oncologic treatment strategy.

Evidence level: Moderate

Recommendation grade: Strong

### How should hepatitis C virus infection be managed in patients diagnosed with HCC?

Saraiya et al.^67^, in a meta-analysis of 24 studies and 1,820 patients, reported a pooled HCC recurrence rate of 24.4% after direct-acting antiviral (DAA) therapy. HCC recurrence rates were comparable between patients treated with DAA and those managed with interferon-based therapy or no antiviral treatment, indicating that DAA treatment does not confer a demonstrable oncologic benefit in patients with active or recently treated HCC.

Although viral eradication with DAAs may improve liver function and, in theory, facilitate subsequent oncologic therapies, this potential advantage must be weighed against evidence of reduced antiviral efficacy in the setting of HCC. In a large real-world analysis of the HCV-TARGET cohort^58^, the authors evaluated 1,457 patients with cirrhosis and complete virologic follow-up. Sustained virologic response rates were 91% in patients without HCC, compared with 84% in those with completely treated HCC and 80% in those with partially treated or untreated HCC. In adjusted analysis, the presence of HCC was independently associated with a significantly lower likelihood of achieving a sustained viral response (SVR) (odds ratio [OR]=0.51; 95%CI 0.33-0.81), whereas tumor treatment status among patients with HCC did not significantly influence SVR. These data indicate that HCC itself, rather than tumor control, is associated with diminished antiviral response. When combined with the substantial competing risk of HCC-related mortality, these findings help explain why the clinical impact of DAA therapy in patients with active HCC is often limited.

These findings support the strategy of deferring DAA therapy until after curative-intent HCC treatment, such as surgical resection or local ablation. Delaying antiviral therapy allows repeated imaging to confirm oncologic response, reduces the likelihood of treating patients with residual or occult disease, and may improve the probability of achieving SVR. On the other hand, in patients listed for liver transplantation, the timing of DAA therapy requires individualized assessment. Considerations include regional waiting times, availability of HCV-positive donor organs, and severity of liver dysfunction^6^.

In contrast, in patients with intermediate or advanced HCC who are not candidates for curative therapy, the likelihood of achieving a complete tumor response is low, and the competing risk of HCC-related mortality is high. In this population, available data are insufficient to demonstrate the benefit of DAA therapy. Therefore, antiviral treatment should not be routinely administered and must be individualized, considering treatment costs, tumor burden, expected survival, liver function, and patient preferences.

### Recommendation 18

In patients with HCV-related HCC, DAA therapy should be offered after a complete response to curative-intent treatments, including surgical resection or local ablation. In candidates for liver transplantation, the timing of DAA therapy should be individualized, considering regional waiting times, availability of HCV-positive donor organs, and the severity of underlying liver dysfunction. In patients who are not candidates for curative treatment, routine DAA therapy is not recommended, and antiviral treatment should be individualized based on treatment costs, tumor burden, expected survival, liver function, and patient preferences. This recommendation reflects current evidence on competing risks rather than a lack of antiviral efficacy.

Evidence level: Low

Recommendation grade: Weak

## DISCUSSION

The management of HCC is intrinsically complex, as it requires simultaneous consideration of tumor burden, liver function, portal hypertension, and patient performance status. Unlike most solid tumors, HCC arises predominantly in the setting of chronic liver disease, demanding close interaction among multiple specialties, including hepatology, radiology, pathology, oncology, surgery, interventional radiology, and transplantation. In this context, standardizing diagnostic and clinical assessment pathways is essential to ensure that all members of the multidisciplinary team share a common language, consistent diagnostic thresholds, and aligned decision-making principles. This consensus directly addresses this need by harmonizing radiological, histopathological, and clinical evaluation strategies, thereby facilitating consistent and transparent care.

Looking ahead, several developments have the potential to transform the clinical investigation and initial management of HCC. Imaging already plays a central role in diagnosis, allowing noninvasive confirmation of HCC in most high-risk patients and avoiding biopsy-related risks. In this setting, artificial intelligence and radiomics are emerging as promising tools capable of extracting high-dimensional imaging features beyond visual assessment^27^ ^,^ ^46^. These approaches may improve lesion characterization, refine risk stratification, predict histological subtypes, and potentially anticipate treatment response. However, their incorporation into routine practice will require rigorous validation, clear demonstration of clinical benefit, and strategies to ensure equitable access to advanced imaging technologies and computational resources.

Advances in pathology also hold promise for improving diagnostic efficiency and consistency. Artificial intelligence-assisted pathology and digital workflows may accelerate reporting, enhance standardization, and support accurate histological and immunohistochemical assessment when tissue diagnosis is required^66^. Nonetheless, these innovations must be implemented alongside investments in training, infrastructure, and quality control to avoid widening existing disparities.

In parallel with advances in imaging and pathology, molecular biomarkers are emerging as a key frontier in HCC risk stratification and early detection. Circulating tumor deoxyribonucleic acid (ct-DNA), circulating tumor cells, and other liquid biopsy-based markers have shown promise for identifying minimal residual disease, detecting early tumor recurrence, and refining prognostic assessment beyond conventional clinical staging. These approaches may enable dynamic, noninvasive monitoring of tumor biology and treatment response, potentially complementing imaging-based surveillance. Liquid biopsy can capture tumor heterogeneity and molecular evolution over time, providing insights that are not obtainable from a single tissue sample and enabling longitudinal assessment of disease activity. However, current evidence remains heterogeneous, and standardized assays, validated thresholds, and prospective studies demonstrating clinical utility are still lacking. Large-scale validation studies are needed to define how these biomarkers should be integrated into existing surveillance algorithms and treatment decision pathways^3^.

Beyond technological innovation, future efforts must prioritize strategies to disseminate diagnostic resources and expand equitable access to care. In many regions, access to cross-sectional imaging, nuclear medicine, antiviral therapy for hepatitis B and C, and structured surveillance programs remain limited. These gaps are particularly concerning because they often disproportionately affect populations with the highest burden of HCC. In Brazil-a large and highly heterogeneous country with marked regional disparities in healthcare infrastructure-, unequal resource allocation can lead to diagnostic delays, inconsistent staging, and unequal access to treatment.

Diagnostic and clinical investigation strategies must be adapted to the sociogeographic realities of each healthcare system. This consensus represents a multidisciplinary effort to translate scientific evidence into pragmatic, context-sensitive recommendations. It should not be viewed as a prescriptive manual, but rather as a decision-support framework designed to guide diagnostic and management pathways across a wide range of clinical environments^76^. Ongoing updates will be essential to incorporate emerging evidence, technological advances, and implementation data, while continuously addressing disparities in access and outcomes.

## CONCLUSION

This multidisciplinary consensus provides evidence-based recommendations for the diagnosis, clinical assessment, and staging of HCC, with emphasis on standardization and clinical applicability. By integrating radiological, histopathological, and clinical parameters, these recommendations aim to support individualized, safe patient care and to serve as a robust framework for future updates that incorporate emerging evidence and evolving clinical practice.

## Data Availability

The datasets generated and/or analyzed during the current study are available from the corresponding author upon reasonable request. The information regarding the investigation, methodology, and data analysis of the article is archived under the authors’ responsibility.

## References

[1] Adamo RG, van der Pol CB, Alabousi M, Lam E, Salameh JP, Abedrabbo N (2025). Diagnostic performance of CT/MRI LI-RADS version 2018 major feature combinations: individual participant data meta-analysis. Radiology.

[2] Al-Muhannadi N, Ansari N, Brahmi U, Satir AA (2011). Differential diagnosis of malignant epithelial tumours in the liver: an immunohistochemical study on liver biopsy material. Ann Hepatol.

[3] Anand N, Wu S, Guo Z, Figueroa MS, Jung L, Li M (2025). Novel blood-based liquid biopsy approaches in hepatocellular carcinoma. JCO Oncol Adv.

[4] Bohte AE, Niet A, Jansen L, Bipat S, Nederveen AJ, Verheij J (2014). Non-invasive evaluation of liver fibrosis: a comparison of ultrasound-based transient elastography and MR elastography in patients with viral hepatitis B and C. Eur Radiol.

[5] Bray F, Laversanne M, Sung H, Ferlay J, Siegel RL, Soerjomataram I (2024). Global cancer statistics 2022: GLOBOCAN estimates of incidence and mortality worldwide for 36 cancers in 185 countries. CA Cancer J Clin.

[6] Bruix J, Castells A, Bosch J, Feu F, Fuster J, Garcia-Pagan JC (1996). Surgical resection of hepatocellular carcinoma in cirrhotic patients: prognostic value of preoperative portal pressure. Gastroenterology.

[7] Cescon M, Colecchia A, Cucchetti A, Peri E, Montrone L, Ercolani G (2012). Value of transient elastography measured with FibroScan in predicting the outcome of hepatic resection for hepatocellular carcinoma. Ann Surg.

[8] Chang TT, Sawhney R, Monto A, Davoren JB, Kirkland JG, Stewart L (2008). Implementation of a multidisciplinary treatment team for hepatocellular cancer at a Veterans Affairs Medical Center improves survival. HPB.

[9] Chernyak V, Fowler KJ, Kamaya A, Kielar AZ, Elsayes KM, Bashir MR (2018). Liver imaging reporting and data system (LI-RADS) version 2018: imaging of hepatocellular carcinoma in at-risk patients. Radiology.

[10] Choi JY, Lee JM, Sirlin CB (2014). CT and MR imaging diagnosis and staging of hepatocellular carcinoma: part II. Extracellular agents, hepatobiliary agents, and ancillary imaging features. Radiology.

[11] Coimbra FJF, Godoy AL, Feitosa H, Pinheiro RN, Boin IFSF, Andraus W (2026). Prevention, surveillance, and early detection of hepatocellular carcinoma: a Brazilian multidisciplinary consensus. Arq Bras Cir Dig.

[12] Cooper GS, Bellamy P, Dawson NV, Desbiens N, Fulkerson WJ, Goldman L (1997). A prognostic model for patients with end-stage liver disease. Gastroenterology.

[13] Corwin MT, Lee AY, Fananapazir G, Loehfelm TW, Sarkar S, Sirlin CB (2018). Nonstandardized terminology to describe focal liver lesions in patients at risk for hepatocellular carcinoma: implications regarding clinical communication. AJR Am J Roentgenol.

[14] Carvalho JR, Villela-Nogueira CA, Perez RM, Portugal FB, Flor LS, Campos MR (2017). Burden of chronic viral hepatitis and liver cirrhosis in Brazil - the Brazilian Global Burden of Disease Study. Ann Hepatol.

[15] Graaf W, Bennink RJ, Veteläinen R, van Gulik TM (2010). Nuclear imaging techniques for the assessment of hepatic function in liver surgery and transplantation. J Nucl Med.

[16] Graaf W, van Lienden KP, van Gulik TM, Bennink RJ (2010). (99m)Tc-mebrofenin hepatobiliary scintigraphy with SPECT for the assessment of hepatic function and liver functional volume before partial hepatectomy. J Nucl Med.

[17] Di Tommaso L, Franchi G, Park YN, Fiamengo B, Destro A, Morenghi E (2007). Diagnostic value of HSP70, glypican 3, and glutamine synthetase in hepatocellular nodules in cirrhosis. Hepatology.

[18] El-Khoueiry AB, Sangro B, Yau T, Crocenzi TS, Kudo M, Hsu C (2017). Nivolumab in patients with advanced hepatocellular carcinoma (CheckMate 040): an open-label, non-comparative, phase 1/2 dose escalation and expansion trial. Lancet.

[19] European Association for the Study of the Liver (2018). EASL Clinical Practice Guidelines: Management of hepatocellular carcinoma. J Hepatol.

[20] Flusberg M, Ganeles J, Ekinci T, Goldberg-Stein S, Paroder V, Kobi M (2017). Impact of a structured report template on the quality of CT and MRI reports for hepatocellular carcinoma diagnosis. J Am Coll Radiol.

[21] Forner A, Vilana R, Ayuso C, Bianchi L, Solé M, Ayuso JR (2008). Diagnosis of hepatic nodules 20 mm or smaller in cirrhosis: Prospective validation of the noninvasive diagnostic criteria for hepatocellular carcinoma. Hepatology.

[22] Francisco FA, Araújo AL, Oliveira JA, Parente DB (2014). Hepatobiliary contrast agents: differential diagnosis of focal hepatic lesions, pitfalls and other indications. Radiol Bras.

[23] Garcia FOB, Garcia RJR, Maurity MP, Nascimento ESM (2024). Hepatectomies: indications and results from a reference hospital in the Brazilian Amazon. Arq Bras Cir Dig.

[24] Ge PL, Du SD, Mao YL (2014). Advances in preoperative assessment of liver function. Hepatobiliary Pancreat Dis Int.

[25] Guo G, Lei Z, Tang X, Ma W, Si A, Yang P (2021). External validation of six liver functional reserve models to predict posthepatectomy liver failure after major resection for hepatocellular carcinoma. J Cancer.

[26] Harding JJ, Abu-Zeinah G, Chou JF, Owen DH, Ly M, Lowery MA (2018). Morbidity, and mortality of bone metastases in advanced hepatocellular carcinoma. J Natl Compr Canc Netw.

[27] He T, Fong JN, Moore LW, Ezeana CF, Victor D, Divatia M (2021). An imageomics and multi-network based deep learning model for risk assessment of liver transplantation for hepatocellular cancer. Comput Med Imaging Graph.

[28] Hiraoka A, Michitaka K, Kumada T, Izumi N, Kadoya M, Kokudo N (2017). Validation and potential of albumin-bilirubin grade and prognostication in a nationwide survey of 46,681 hepatocellular carcinoma patients in Japan: the need for a more detailed evaluation of hepatic function. Liver Cancer.

[29] Ho MC, Hasegawa K, Chen XP, Nagano H, Lee YJ, Chau GY (2016). Surgery for intermediate and advanced hepatocellular carcinoma: a consensus report from the 5th Asia-Pacific Primary Liver Cancer Expert Meeting (APPLE 2014). Liver Cancer.

[30] Hu S, Gan W, Qiao L, Ye C, Wu D, Liao B (2021). A new prognostic algorithm predicting HCC recurrence in patients with Barcelona clinic liver cancer stage B who received PA-TACE. Front Oncol.

[31] Imajo K, Honda Y, Kobayashi T, Nagai K, Ozaki A, Iwaki M (2022). Direct comparison of US and MR elastography for staging liver fibrosis in patients with nonalcoholic fatty liver disease. Clin Gastroenterol Hepatol.

[32] Jin YJ, Lee HC, Lee D, Shim JH, Kim KM, Lim YS (2012). Role of the routine use of chest computed tomography and bone scan in staging workup of hepatocellular carcinoma. J Hepatol.

[33] Johnson PJ, Berhane S, Kagebayashi C, Satomura S, Teng M, Reeves HL (2015). Assessment of liver function in patients with hepatocellular carcinoma: a new evidence-based approach-the ALBI grade. J Clin Oncol.

[34] Kabbach G, Assi HA, Bolotin G, Schuster M, Lee HJ, Tadros M (2015). Hepatobiliary tumors: update on diagnosis and management. J Clin Transl Hepatol.

[35] Kanneganti M, Marrero JA, Parikh ND, Kanwal F, Yokoo T, Mendiratta-Lala M (2022). Clinical outcomes of patients with Liver Imaging Reporting and Data System 3 or Liver Imaging Reporting and Data System 4 observations in patients with cirrhosis: A systematic review. Liver Transpl.

[36] Kim H, Jang M, Park YN (2020). Histopathological variants of hepatocellular carcinomas: an update according to the 5th edition of the WHO Classification of Digestive System Tumors. J Liver Cancer.

[37] Kim TH, Kim SY, Tang A, Lee JM (2019). Comparison of international guidelines for noninvasive diagnosis of hepatocellular carcinoma: 2018 update. Clin Mol Hepatol.

[38] Lee PC, Chao Y, Chen MH, Lan KH, Lee IC, Hou MC (2020). Risk of HBV reactivation in patients with immune checkpoint inhibitor-treated unresectable hepatocellular carcinoma. J Immunother Cancer.

[39] Lee S, Kim SS, Roh YH, Choi JY, Park MS, Kim MJ (2020). Diagnostic performance of CT/MRI liver imaging reporting and data system v2017 for hepatocellular carcinoma: a systematic review and meta-analysis. Liver Int.

[40] Lee SH, Yim SY, Shim JJ, Lee JS, Hoshida Y (2019). Hepatocellular carcinoma: translational precision medicine approaches.

[41] Lin F, Abdallah H, Meschter S (2004). Diagnostic utility of CD10 in differentiating hepatocellular carcinoma from metastatic carcinoma in fine-needle aspiration biopsy (FNAB) of the liver. Diagn Cytopathol.

[42] Liu Y, Veeraraghavan V, Pinkerton M, Fu J, Douglas MW, George J (2021). Viral biomarkers for hepatitis B virus-related hepatocellular carcinoma occurrence and recurrence. Front Microbiol.

[43] Loy LM, Low HM, Choi JY, Rhee H, Wong CF, Tan CH (2022). Variant hepatocellular carcinoma subtypes according to the 2019 WHO classification: an imaging-focused review. AJR Am J Roentgenol.

[44] Lu RC, She B, Gao WT, Ji YH, Xu DD, Wang QS (2019). Positron-emission tomography for hepatocellular carcinoma: Current status and future prospects. World J Gastroenterol.

[45] Machairas N, Papaconstantinou D, Tsilimigras DI, Moris D, Prodromidou A, Paspala A (2019). Comparison between robotic and open liver resection: a systematic review and meta-analysis of short-term outcomes. Updates Surg.

[46] Malik S, Das R, Thongtan T, Thompson K, Dbouk N (2024). AI in hepatology: revolutionizing the diagnosis and management of liver disease. J Clin Med.

[47] Marrero JA, Kulik LM, Sirlin CB, Zhu AX, Finn RS, Abecassis MM (2018). Diagnosis, staging, and management of hepatocellular carcinoma: 2018 practice guidance by the American Association for the Study of Liver Diseases. Hepatology.

[48] McGlynn KA, Petrick JL, El-Serag HB (2021). Epidemiology of hepatocellular carcinoma. Hepatology.

[49] National Comprehensive Cancer Network (2022). Hepatobiliary cancers.

[50] Nault JC, Boubaya M, Wartski M, Dohan A, Pol S, Pop G (2025). [18F]fluorodeoxyglucose and [18F]fluorocholine PET-CT for staging optimisation and treatment modification in hepatocellular carcinoma (PET-HCC01): a prospective multicentre study. Lancet Gastroenterol Hepatol.

[51] Nguyen T, Phillips D, Jain D, Torbenson M, Wu TT, Yeh MM (2015). Comparison of 5 immunohistochemical markers of hepatocellular differentiation for the diagnosis of hepatocellular carcinoma. Arch Pathol Lab Med.

[52] Nie C, Vaska M, Wong JK, Bathe OF, Przybojewski S, Burak KW (2025). Tumor seeding with needle biopsy of hepatocellular carcinoma: a systematic review. Am J Gastroenterol.

[53] Oxenberg J, Papenfuss W, Esemuede I, Attwood K, Simunovic M, Kuvshinoff B (2015). Multidisciplinary cancer conferences for gastrointestinal malignancies result in measureable treatment changes: a prospective study of 149 consecutive patients. Ann Surg Oncol.

[54] PDQ® Adult Treatment Editorial Board (2023). PDQ adult primary liver cancer treatment.

[55] Pinato DJ, Sharma R, Allara E, Yen C, Arizumi T, Kubota K (2017). The ALBI grade provides objective hepatic reserve estimation across each BCLC stage of hepatocellular carcinoma. J Hepatol.

[56] Pomfret EA, Washburn K, Wald C, Nalesnik MA, Douglas D, Russo M (2010). Report of a national conference on liver allocation in patients with hepatocellular carcinoma in the United States. Liver Transpl.

[57] Quireze C, Coelho FF, Lima AS, Marques HP, Palavecino M, Pawlik T (2025). Complications after hepatectomy. Arq Bras Cir Dig.

[58] Radhakrishnan K, Di Bisceglie AM, Reddy KR, Lim JK, Levitsky J, Hassan MA (2019). Treatment status of hepatocellular carcinoma does not influence rates of sustained virologic response: an HCV-TARGET analysis. Hepatol Commun.

[59] Rassam F, Olthof PB, Richardson H, van Gulik TM, Bennink RJ (2019). Practical guidelines for the use of technetium-99m mebrofenin hepatobiliary scintigraphy in the quantitative assessment of liver function. Nucl Med Commun.

[60] Reig M, Forner A, Rimola J, Ferrer-Fàbrega J, Burrel M, Garcia-Criado Á (2022). BCLC strategy for prognosis prediction and treatment recommendation: The 2022 update. J Hepatol.

[61] Roayaie S, Jibara G, Tabrizian P, Park JW, Yang J, Yan L (2015). The role of hepatic resection in the treatment of hepatocellular cancer. Hepatology.

[62] Rodriguez-Alvarez F, Mota-Ayala BZ, Villavicencio-Martínez R, Kauffman-Ortega E, Téllez-Morán LS, Castro-Narro G (2024). Limited utility of routine bone scintigraphy in the staging of patients with hepatocellular carcinoma: A cross-sectional study. Ann Hepatol.

[63] Roskams T, Kojiro M (2010). Pathology of early hepatocellular carcinoma: conventional and molecular diagnosis. Semin Liver Dis.

[64] Salgia R, Mendiratta V (2021). The multidisciplinary management of hepatocellular carcinoma. Clin Liver Dis.

[65] Sangiovanni A, Manini MA, Iavarone M, Romeo R, Forzenigo LV, Fraquelli M (2010). The diagnostic and economic impact of contrast imaging techniques in the diagnosis of small hepatocellular carcinoma in cirrhosis. Gut.

[66] Sanyal P, Biswas D, Mitra S (2025). Artificial intelligence in liver pathology: precision histology for accurate diagnoses. J Clin Exp Hepatol.

[67] Saraiya N, Yopp AC, Rich NE, Odewole M, Parikh ND, Singal AG (2018). Systematic review with meta-analysis: recurrence of hepatocellular carcinoma following direct-acting antiviral therapy. Aliment Pharmacol Ther.

[68] Schulze K, Imbeaud S, Letouzé E, Alexandrov LB, Calderaro J, Rebouissou S (2015). Exome sequencing of hepatocellular carcinomas identifies new mutational signatures and potential therapeutic targets. Nat Genet.

[69] Sempoux C, Jibara G, Ward SC, Fan C, Qin L, Roayaie S (2011). Intrahepatic cholangiocarcinoma: new insights in pathology. Semin Liver Dis.

[70] Sempoux C, Kakar S, Kondo F, Schirmacher P, WHO Classification of Tumours Editorial Board (2019). WHO classification of tumours of the digestive system.

[71] Serper M, Taddei TH, Mehta R, D’Addeo K, Dai F, Aytaman A (2017). Association of provider specialty and multidisciplinary care with hepatocellular carcinoma treatment and mortality. Gastroenterology.

[72] Sun X, Hu D, Yang Z, Liu Z, Wang J, Chen J (2020). Baseline HBV loads do not affect the prognosis of patients with hepatocellular carcinoma receiving anti-programmed cell death-1 immunotherapy. J Hepatocell Carcinoma.

[73] Teng W, Chang TT, Yang HI, Peng CY, Su CW, Su TH (2021). Risk scores to predict HCC and the benefits of antiviral therapy for CHB patients in gray zone of treatment guidelines. Hepatol Int.

[74] Torbenson MS, Ng IOL, Park YN, Roncalli M, Sakamoto M, WHO Classification of Tumours Editorial Board (2019). Digestive system tumours. WHO classification of tumours series.

[75] Torzilli G, Belghiti J, Kokudo N, Takayama T, Capussotti L, Nuzzo G (2013). A snapshot of the effective indications and results of surgery for hepatocellular carcinoma in tertiary referral centers: is it adherent to the EASL/AASLD recommendations?: an observational study of the HCC East-West study group. Ann Surg.

[76] Tustumi F, Calthorpe L, Coimbra FJF, Alseidi A (2026). Reviews, expert opinions, consensus statements, position papers, protocols, and evidence-based guidelines: what are their roles in clinical practice?. Arq Bras Cir Dig.

[77] Tustumi F, Coelho FF, Magalhães DP, Silveira S, Jeismann VB, Fonseca GM (2023). Treatment of hepatocellular carcinoma with macroscopic vascular invasion: A systematic review and network meta-analysis. Transplant Rev.

[78] van der Pol CB, Lim CS, Sirlin CB, McGrath TA, Salameh JP, Bashir MR (2019). Accuracy of the liver imaging reporting and data system in computed tomography and magnetic resonance image analysis of hepatocellular carcinoma or overall malignancy-a systematic review. Gastroenterology.

[79] van Dievoet MA, Eeckhoudt S, Stephenne X (2020). Primary hemostasis in chronic liver disease and cirrhosis: what did we learn over the past decade?. Int J Mol Sci.

[80] Villar LM, Milagres FAP, Lampe E, Cruz HM, Scalioni LP, Magalhães MAFM (2018). Determination of hepatitis B, C and D prevalence among urban and Amerindian populations from the Eastern Brazilian Amazon: a cross sectional study. BMC Infect Dis.

[81] Vitale A, Burra P, Frigo AC, Trevisani F, Farinati F, Spolverato G (2015). Survival benefit of liver resection for patients with hepatocellular carcinoma across different Barcelona Clinic Liver Cancer stages: a multicentre study. J Hepatol.

[82] Wald C, Russo MW, Heimbach JK, Hussain HK, Pomfret EA, Bruix J (2013). New OPTN/UNOS policy for liver transplant allocation: standardization of liver imaging, diagnosis, classification, and reporting of hepatocellular carcinoma. Radiology.

[83] Wang L, Vuolo M, Suhrland MJ, Schlesinger K (2006). HepPar1, MOC-31, pCEA, mCEA and CD10 for distinguishing hepatocellular carcinoma vs. metastatic adenocarcinoma in liver fine needle aspirates. Acta Cytol.

[84] Wang Y, Guan Y, Abbas AR, Sano Y, Jain S, Lu S (2025). Molecular subtypes of hepatocellular carcinoma linked to liver cell lineages and clinical outcomes of combination immunotherapy. Cell Rep Med.

[85] Xu HW, Liu F, Li HY, Wei YG, Li B (2018). Outcomes following laparoscopic versus open major hepatectomy for hepatocellular carcinoma in patients with cirrhosis: a propensity score-matched analysis. Surg Endosc.

[86] Xu L, Gao H, Huang J, Wang H, Zhou Z, Zhang Y (2015). Antiviral therapy in the improvement of survival of patients with hepatitis B virus-related hepatocellular carcinoma treated with sorafenib. J Gastroenterol Hepatol.

[87] Yamamoto M, Kobayashi T, Hashimoto M, Kuroda S, Kawaoka T (2021). Significance of liver resection for intermediate stage hepatocellular carcinoma according to subclassification. BMC Cancer.

[88] Yang JD, Hainaut P, Gores GJ, Amadou A, Plymoth A, Roberts LR (2019). A global view of hepatocellular carcinoma: trends, risk, prevention and management. Nat Rev Gastroenterol Hepatol.

[89] Yilma M, Xu RH, Saxena V, Muzzin M, Tucker LY, Lee J (2024). Survival outcomes among patients with hepatocellular carcinoma in a large integrated US health system. JAMA Netw Open.

[90] Yin Y, Liu J, Sun R, Liu X, Zhou Z, Zhang H (2023). Exploring the efficacy of 18F-FDG PET/CT in hepatocellular carcinoma diagnosis: role of Ki-67 index and tumor differentiation. Abdom Radiol (NY).

[91] Yoneda N, Sato Y, Kitao A, Ikeda H, Sawada-Kitamura S, Miyakoshi M (2011). Epidermal growth factor induces cytokeratin 19 expression accompanied by increased growth abilities in human hepatocellular carcinoma. Lab Invest.

[92] Yopp AC, Mansour JC, Beg MS, Arenas J, Trimmer C, Reddick M (2014). Establishment of a multidisciplinary hepatocellular carcinoma clinic is associated with improved clinical outcome. Ann Surg Oncol.

[93] Zhang YN, Fowler KJ, Boehringer AS, Montes V, Schlein AN, Covarrubias Y (2022). Comparative diagnostic performance of ultrasound shear wave elastography and magnetic resonance elastography for classifying fibrosis stage in adults with biopsy-proven nonalcoholic fatty liver disease. Eur Radiol.

[94] Zhou HY, Luo Y, Chen WD, Gong GZ (2015). Hepatitis B virus mutation may play a role in hepatocellular carcinoma recurrence: A systematic review and meta-regression analysis. J Gastroenterol Hepatol.

[95] Zhou J, Sun H, Wang Z, Cong W, Wang J, Zeng M (2020). Guidelines for the Diagnosis and Treatment of Hepatocellular Carcinoma (2019 Edition). Liver Cancer.

